# Objectivization study of acupuncture *Deqi* and brain modulation mechanisms: a review

**DOI:** 10.3389/fnins.2024.1386108

**Published:** 2024-05-03

**Authors:** Zhen Zhong, Lin Yao, Yan-Ze Liu, Yu Wang, Min He, Meng-Meng Sun, Hai-Peng Huang, Shi-Qi Ma, Hai-Zhu Zheng, Meng-Yuan Li, Xin-Yu Zhang, De-Yu Cong, Hong-Feng Wang

**Affiliations:** ^1^College of Acupuncture and Massage, Changchun University of Chinese Medicine, Changchun, Jilin, China; ^2^Institute of Acupuncture and Massage, Northeast Asian Institute of Traditional Chinese Medicine, Changchun University of Chinese Medicine, Changchun, Jilin, China; ^3^Acupuncture and Tuina Center, The 3rd Affiliated Hospital of Changchun University of Chinese Medicine, Changchun, China; ^4^Northeast Asian Institute of Traditional Chinese Medicine, Changchun University of Chinese Medicine, Changchun, China; ^5^Department of Tuina, Traditional Chinese Medicine Hospital of Jilin Province, Changchun, China

**Keywords:** acupuncture, *Deqi*, objectivization, functional magnetic resonance imaging, brain center

## Abstract

*Deqi* is an important prerequisite for acupuncture to achieve optimal efficacy. Chinese medicine has long been concerned with the relationship between *Deqi* and the clinical efficacy of acupuncture. However, the underlying mechanisms of *Deqi* are complex and there is a lack of systematic summaries of objective quantitative studies of *Deqi*. Acupuncture *Deqi* can achieve the purpose of treating diseases by regulating the interaction of local and neighboring acupoints, brain centers, and target organs. At local and neighboring acupoints, *Deqi* can change their tissue structure, temperature, blood perfusion, energy metabolism, and electrophysiological indicators. At the central brain level, *Deqi* can activate the brain regions of the thalamus, parahippocampal gyrus, postcentral gyrus, insular, middle temporal gyrus, cingulate gyrus, etc. It also has extensive effects on the limbic-paralimbic-neocortical-network and default mode network. The brain mechanisms of *Deqi* vary depending on the acupuncture techniques and points chosen. In addition, *Deqi* 's mechanism of action involves correcting abnormalities in target organs. The mechanisms of acupuncture *Deqi* are multi-targeted and multi-layered. The biological mechanisms of Deqi are closely related to brain centers. This study will help to explore the mechanism of *Deqi* from a local-central-target-organ perspective and provide information for future clinical decision-making.

## 1 Introduction to the basic theories of acupuncture and *Deqi*

Acupuncture belongs to the category of complementary and alternative medicine therapies and has been around for more than 3,000 years (Han and Ho, 2011). Its efficacy is gradually being recognized worldwide (Lederer et al., 2018; Allen et al., 2022). In traditional Chinese medicine theory, the occurrence of disease is related to the poor functioning of “*Qi”* (Filshie et al., 2016) (flowing energy or information) in the meridian system (Ziegler, 1999; Rong et al., 2011). The meridian system consists of 12 meridians, which are used for “qi” and blood flow (Deadman et al., 2001; Takamoto et al., 2013). Visualization techniques can provide prima facie evidence for the existence of meridians from an anatomical perspective (Yang C. et al., 2015; Xiong et al., 2020). Early studies have shown that injection of tracers at acupoints produces trajectories that highly correspond to the distribution of meridians (Wu et al., 1990, 1994; de Vernejoul et al., 1992; Chen et al., 1993; Kovacs et al., 1996, 2000; Li et al., 2012). In recent years, such phenomena have also been observed in humans (Dimitrov et al., 2021; Li T. et al., 2021). A growing body of studies are devoted to explaining the nature of the meridians, including neural-fluid-low-flow resistance, fascial neuromodulation, connective tissue matrices, and low-water-resistance channels (Yang and Han, 2015; Zhang et al., 2015, 2019; Bianco, 2019; Yonghong et al., 2020). In addition to the material properties of meridians, their functional, temporal, and cultural properties are gradually being emphasized (Ye et al., 2022). The meridian system is considered to be an important channel that connects the surface of the body to the internal organs (Yung, 2005; Neumann et al., 2023). There are many acupoints distributed on the meridians, which are the reflection points of diseases (Melzack et al., 1977; Furlan et al., 2005). Stimulation of different acupoints on the body surface can deal with diseases of different systems. The physiological basis of acupoints has been studied for a long time, and studies have shown that the structure of acupoints is related to the nervous system, blood vessels, and muscles (Kuo et al., 2004; Lee et al., 2008; Zhao, 2008; Silberstein, 2012). The WHO Western Pacific Regional Office developed the WHO Standard Acupuncture Point Locations in 2008 (World Health Organization, Regional Office for the Western Pacific, 2008). It gives a detailed description of the number and location of acupoints. Different acupoints have different sizes (Molsberger et al., 2012; Rong et al., 2013). Some studies have shown that the area of commonly used acupoints ranges from 2.7 to 41.4 cm^2^ (Molsberger et al., 2012). Meridians and acupoints are affected by a variety of factors and there are certain individual differences. For example, the size and location of the meridians and acupoints may vary depending on the patient's body type, pathophysiologic state, and the doctor's needling angle.

Acupuncture can be used to balance and restore the body's energy (called “*Qi”* in Chinese medicine) by stimulating acupoints (Zhou and Benharash, 2014; Chen H. et al., 2019). When acupuncture stimulation reaches a certain intensity, *Deqi* (acquisition of “*Qi”*) is produced. *Deqi*, also known as needle sensation, is an important parameter in the study and evaluation of acupuncture efficacy (Zhao et al., 2017; Hu et al., 2019; Yang et al., 2020; Sun et al., 2021). *Deqi* is a complex physiological state involving nerve fibers at all levels, muscles, connective tissue, etc. (Langevin et al., 2001; Leung et al., 2006; Hui et al., 2007; Jung et al., 2016). *Deqi* on the part of the patient shows numbness, dull aches, heaviness, soreness, fullness and so on (Yuan et al., 2013b; Ren et al., 2015). The acupuncturists feel increased resistance under the needle during *Deqi* (Chen S. et al., 2013; Yin et al., 2015). In summary, *Deqi* is a composite of sensations obtained by the patient and the acupuncturist during acupuncture interventions (Kong et al., 2007).

*Deqi* is reported to directly affect the clinical efficacy of acupuncture (Yuan et al., 2013a; Zhao et al., 2017; Zhang et al., 2020), so the objectivization of acupuncture *Deqi* is a major challenge (Chen S. et al., 2013). Previously, *Deqi* objectification studies have focused on descriptive analyses of the production and intensity of needle sensation by scales (Vincent et al., 1989; Park et al., 2002; Kong et al., 2005, 2007). With the development of modern science and technology, it has become possible to explore the biological mechanisms of acupuncture *Deqi*. Examples include functional magnetic resonance imaging (fMRI) (Shi et al., 2016; Sun et al., 2021; Yoon et al., 2023), electroencephalography (EEG) (Lee et al., 2017; Si et al., 2021), multichannel functional near infrared spectroscopy (fNIRS) (Sun et al., 2021), electromyography (Lu et al., 2021), and electrocardiography (Huang et al., 2012c). It has been found that *Deqi* not only increases local blood flow and skin temperature (Zhu et al., 2013) and modulates disease states in target organs, but also produces changes at the brain level (Zhang et al., 2021). Among them, the effect of *Deqi* on brain centers is of great interest to researchers.

Therefore, the present study explored the regulatory mechanism of acupuncture *Deqi* from three aspects: the local material basis of the acupuncture point area, the central brain effect, and the target organ. This review systematically combs through the progress of objective and quantitative research on *Deqi* and provides an in-depth analysis of its research characteristics for future research.

## 2 Current understanding and research focusing on the quantitation of acupuncture *Deqi*

### 2.1 Clinical research

In recent years, there has been increasing clinical evidence that the deqi sensation by acupuncture can influence the effectiveness of treating disease. In cardiovascular diseases, acupuncture *Deqi* can improve the insulin resistance status, reduce blood lipids and serum TNF-α levels in hypertensive patients (Zhang et al., 2016). A study of 300 patients with coronary artery disease showed that acupuncture *Deqi* was more effective (You et al., 1992). Li et al. (2014b) found that *Deqi* sensations appearing early can reduce the incidence of post-stroke spasticity. For gynecological disorders, acupuncture *Deqi* can improve the Kuppermann score for menopausal syndrome and regulate reproductive endocrine hormone levels (Lin et al., 2017). Several studies of acupuncture for primary dysmenorrhea have shown a positive correlation between the speed of *Deqi* sensation production and the speed of clinical effect (Li et al., 2014a; Wang et al., 2018). The intensity of *Deqi* was also positively correlated with the degree of pain relief (Shi et al., 2014; Hu et al., 2019). In gastrointestinal disorders, Sun et al. (2021) found that the Nepean Dyspepsia Symptom Index (NDSI) scores of patients with functional dyspepsia were substantially reduced and improved dyspepsia symptoms after acupuncture *Deqi*. In addition, acupuncture *Deqi* has good clinical efficacy for pain-based disorders. Several studies have confirmed that acupuncture *Deqi* relieves pain, improves knee swelling, and restores motor function in patients with Knee Osteoarthritis (Chen R. et al., 2013; Spaeth et al., 2013). Studies have shown that acupuncture *Deqi* treatment for patients with neck pain is able to reduce NPQ neck pain scale scores (Xu and Fu, 2014), and their acupoint pressure pain thresholds are lower (Duan et al., 2018).

In clinical practice, the *Deqi* scales are frequently used to evaluate the clinical efficacy of acupuncture *Deqi*. The Vincent questionnaire (Vincent et al., 1989), also known as Acupuncture Sensation Scale (ASS), developed by Vincent C An et al in 1989, is the primary self-made needle sensation scale with the characteristics of traditional Chinese medicine in the world. It condenses 20 entries to describe “needle sensation” by referring to the form of McGill Questionnaire (MGQ) and the experience of clinical acupuncturists. The Park Questionnaire (Park et al., 2002), also known as the Korean version of the needle sense scale, was formed by translating the Vincent questionnaire into Korean in 2002 and adding 5 new entries on this basis. These two scales are highly original self-made acupuncture scales, but they do not distinguish pain from “needle sensation”, so they are not widely used in later studies. With the deepening of the study of *Deqi*, in 2005, Kong J and others established Subjective Acupuncture Sensation Scale (SASS)in 2005 (Kong et al., 2005), which introduced emotional assessment words for the first time and graded each sensation. In 2007, this table was further improved to form MGH Acupuncture Sensation Scale(MASS) (Kong et al., 2007), including the main table and two schedules. The needle sensation intensity was recorded in the main table according to the 10cm scale of the Visual Analog Scales (VAS), and the attached tables were Acupuncture Sensation Spreading Scale and Mood Scale. Based on the wide application of the scale, C-MMASS and the Japanese MASS were formed after translation and modification. In addition, there are studies that distinguish *Deqi* sensation of pain sensation, such as Macpherson questionnaire and Southampton Needling Sensation Questionnaire (SNSQ). How to differentiate between pain and *Deqi* sensation has been controversial. A questionnaire showed that nine sensations such as sharp and burning were classified as pain, and seven sensations such as aching, dull, heavy and numb were classified as *Deqi* (MacPherson and Asghar, 2006). Most acupuncturists consider dull pain to be *Deqi*, while sharp pain as a noxious stimulus is not *Deqi* (Hui et al., 2011). However, since *Deqi* is affected by sensory, emotional, and cognitive factors, it is difficult to quantify *Deqi* by scale descriptions alone (Lin et al., 2013).

#### 2.1.1 Local mechanisms of acupuncture Deqi

Localized acupoints are the starting sites for the occurrence of the *Deqi* sensation, and it is crucial to study the *Deqi* generation mechanism. Therefore, the study explored the generation mechanism of *Deqi* from localized acupoints. Researchers have already conducted in-depth studies on issues related to local changes in acupuncture *Deqi* using neurophysiology, anatomy, histochemistry, biomacromolecules, biology, light, electricity, magnetism, and other techniques. The main components of the study include the application of the detector to study the tissue structure, temperature, muscle tension, blood perfusion volume, transcutaneous CO_2_ emission, and volt-ampere characteristics of local and nearby acupoints. Observe the influence of before and after *Deqi*, acupuncture, and sham acupuncture, different manipulations on the above indexes. Most of the selected acupoints are commonly used in the limbs, and the frequency from high to low is ST36, LI4, PC6, SP6, and CV4 (Table 1).

**Table 1:** Study on the effect of acupuncture *Deqi* on local and nearby acupoints.

**Acupoints**	**Research contents**	**Instruments**	**Findings**	**References**
ST36	AC	Ultrasound diagnostic equipment	***The areas of DQ sensation: myofascial and fascial***.	Wu et al., 2017
	PA-AA	Ultrasound diagnostic equipment	Muscle tension↑.	Ren et al., 2013
	DQ-SA/ DM	Infrared thermal camera	***DQ: the skin temperature increased continually and then decreased in the last phase***.	Huang et al., 2013
			***SA: the skin temperature of ST36 decreased in the first 5 minutes and then increased gradually***.	
			Lifting-thrusting>twirling.	
	PA-AA/ DM	Laser Doppler Scanner	***Lifting-thrusting:*** microvascular perfusion↑;	Huang et al., 2012c
			***Twirling:*** microvascular perfusion↓.	
	*DQ-NR*	Volt-ampere characteristic detection system	***DQ: range volt-ampere area, reduced range volt-ampere area and inertia area↓;***	Zhou et al., 2011
			***NR: range volt-ampere area, reduced range volt-ampere area and inertia area↑***.	
	*DQ-NR*	Physiological recorder	***DQ>NR: muscle contractile force***.	Deng and Zhou, 2010
	DQ-SA	Color Doppler ultrasound instrument	***DQ>SA: anterior tibial artery blood flow***.	Zhang et al., 2009
ST36, LI4	PA-AA	Laser Doppler Perfusion Imaging	DQ felt numbness, heaviness, swelling, and soreness, the skin blood flow significantly increased.	Tian et al., 2014
LI4	DQ-SA	Infrared thermography	***DQ: skin temperature↑;***	Agarwal-Kozlowski et al., 2009
			***SA: skin temperature↓***.	
LI4, LI1, LI3, LI5	DQ-SA	Speckle Laser Blood Flow Scanning Technology	***DQ: blood perfusion volume↑;***	Huang et al., 2012b
			***SA: blood perfusion volume↓***.	
LI4, LI11	PA-AA	Laser Doppler flowmetry	***DQ LI4: felt soreness and numbness;***	Kuo et al., 2004
			***DQ LI11: blood flow and palm temperature↑***.	
PC6	PA-AA DQ-SA	Carbon dioxide measuring instrument	The release of transcutaneous CO_2_ emission at the acupoint on the same meridian↑.	Huang and Cheng, 2013
	DQ-SA	Laser Doppler blood perfusion imager	***DQ:the amount of microvascular perfusion↓;***	Huang et al., 2012a
			***SA: no changes***.	
	AC	Ultrasonographic transducer	No association between the number of nerve contacts and DQ.	Streitberger et al., 2007
SP6, CV4	PA-AA	Infrared thermal camera	Skin temperature↑.	Wu et al., 2016
SP6	PA-AA	Infrared thermal camera	Skin temperature of ipsilateral SP6 and SP10↑.	Yang et al., 2014
CV4,LI10	AC	Color Doppler ultrasound instrument	***The areas of DQ sensation: myofascial and fascial***.	Yang L. et al., 2015
KI3	PA-AA	Thermography	Body surface temperature of the right leg↓.	Pólito and Ferreira, 2013
NA	PA-AA/ DM	EMG signal instrument	***DQ reinforcing method: volume pulse wave and pulse rate↓;***	Liu et al., 2005
			***DQ reducing method: volume pulse wave and pulse rate↑***.	

AC, Anatomical characteristics; PA, Before Acupuncture; AA, after Acupuncture; DQ, Deqi; SA, Sham Acupuncture; DM, Different manipulation; NR, no reaction;↑: raise;↓: decline.

Studies have shown that the structural basis of *Deqi* includes numerous muscle fibers, nerve terminals, deep acupoint receptors, blood vessels, and nerve structures of blood vessel walls. The basis of *Deqi* is muscle contraction, nerve electrical conduction, local active substance release, and information transmission between cells caused by acupuncture (Yu et al., 1997). However, the acupoint material basis of electro-acupuncture sensation is different from manual-acupuncture sensation. The former is mainly deep pain receptors and skin pain receptors, while the latter is mainly deep pain receptors (Dong et al., 2007a,b). Based on the study of ST36, it is found that stratifying acupuncture at ST36 can be *Deqi* in every layer, but the middle group has a stronger sense of acupuncture than the shallow and deep groups (Zhao and Wang, 2016).

Existing studies based on pathological conditions have shown that acupuncture *Deqi* can alter pain thresholds and temperatures at disease-specific acupoints. More research is based on physiological conditions, five studies using the ultrasonic technique to study the structural characteristics of the acupoint area (Streitberger et al., 2007; Zhang et al., 2009; Ren et al., 2013; Yang L. et al., 2015; Wu et al., 2017). The results further confirmed that the main tissue structure of acupuncture was muscle fascia, and it could enhance muscle tension. High-frequency ultrasound technology can measure the specific position of the needle tip and accurately display the anatomical structure near the acupoint, which is a reliable method for objective analysis of the relationship between *Deqi* and anatomical structure.

Five studies have used an infrared thermal imager to observe the temperature (Agarwal-Kozlowski et al., 2009; Huang et al., 2013; Pólito and Ferreira, 2013; Yang et al., 2014; Wu et al., 2016). The results showed that acupuncture *Deqi* can significantly increase the temperature of the acupoint area and nearby skin surface, and the temperature rise caused by lifting-thrusting was significantly higher than that by twirling manipulation, while the false acupuncture had the opposite effect. Five studies have used the doppler blood flow meter to observe the blood perfusion (Kuo et al., 2004; Huang et al., 2012a,b,c; Tian et al., 2014). The results showed that acupuncture *Deqi* would cause changes in blood circulation on local and nearby acupoints, with the increase of blood perfusion reported more frequently, while needling without meridians or acupuncture without *Deqi* would have the opposite changes.

Another study has studied the transcutaneous CO_2_ emission (Huang and Cheng, 2013). The results showed that the change in CO_2_ emission through the skin was consistent with the increase in blood flow and skin temperature, which indicated that the energy metabolism indexes of the local and nearby acupoints can objectively reflect *Deqi*. Three studies have studied electrophysiological indexes (Liu et al., 2005; Deng and Zhou, 2010; Zhou et al., 2011). The results showed that acupuncture *Deqi* could change the indexes of muscle tension, volt-ampere characteristic, and myoelectric signal on local and nearby acupoints.

#### 2.1.2 Brain mechanisms of acupuncture Deqi

**Figure 1:**
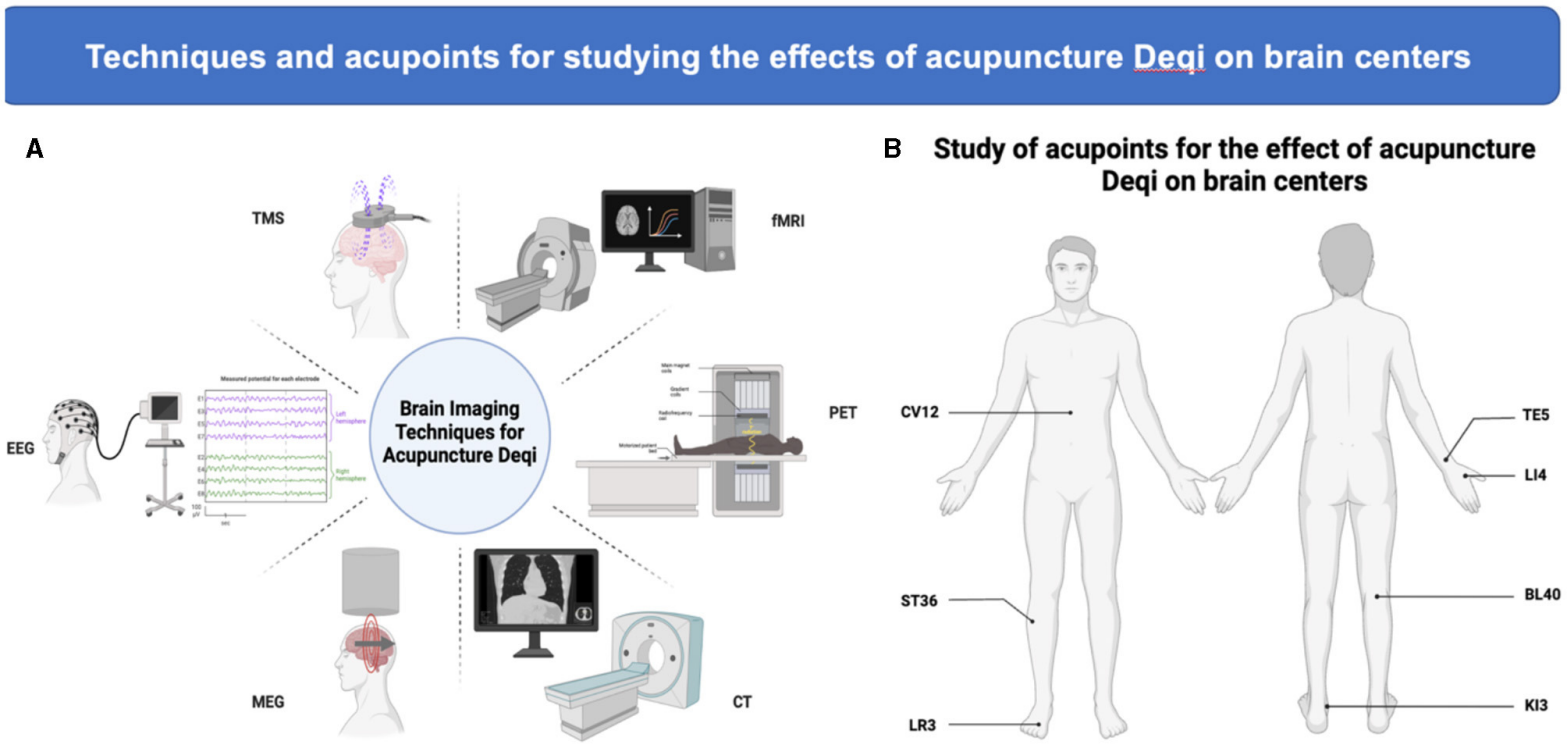
Techniques and acupoints for studying the effects of acupuncture *Deqi* on brain centers. **(A)** Commonly used techniques for the detection of brain centers. **(B)** High-frequency acupoints. Summarize and label the acupoints that were used more frequently than twice in the studies (created with BioRender.com).

Acupuncture at local acupoints can cause sensation such as soreness, numbness, heaviness, and swelling in subjects, which must be transmitted to the brain in order to be perceived. Studies have shown that one of the important ways in which acupuncture regulates the state of the body is mediated through the central system of the brain. After needling acupoints, the stimulus travels from the afferent pathway to the center of the brain for information integration, and then acts on the target organ in a wide range of forms (Torres-Rosas et al., 2014; Liu et al., 2020, 2021; Li N. et al., 2021; Ulloa, 2021; Li et al., 2022). However the mechanisms of information integration and efferent pathways in the brain have not been fully elucidated. Therefore, studying the effect of acupuncture on brain function can help reveal the mechanism of producing acupuncture. In recent years, with the rapid development of brain function detection technology, the detection methods used in the brain science of *Deqi* include electroencephalogram (EEG), transcranial magnetic stimulation (TMS), computed tomography (CT), Magnetoencephalography (MEG), positron emission tomography (PET), functional magnetic resonance imaging (fMRI) and so on. fMRI is the most widely used technical tool for the relationship between acupuncture *Deqi* and brain function (Figure 1).

**Figure 2:**
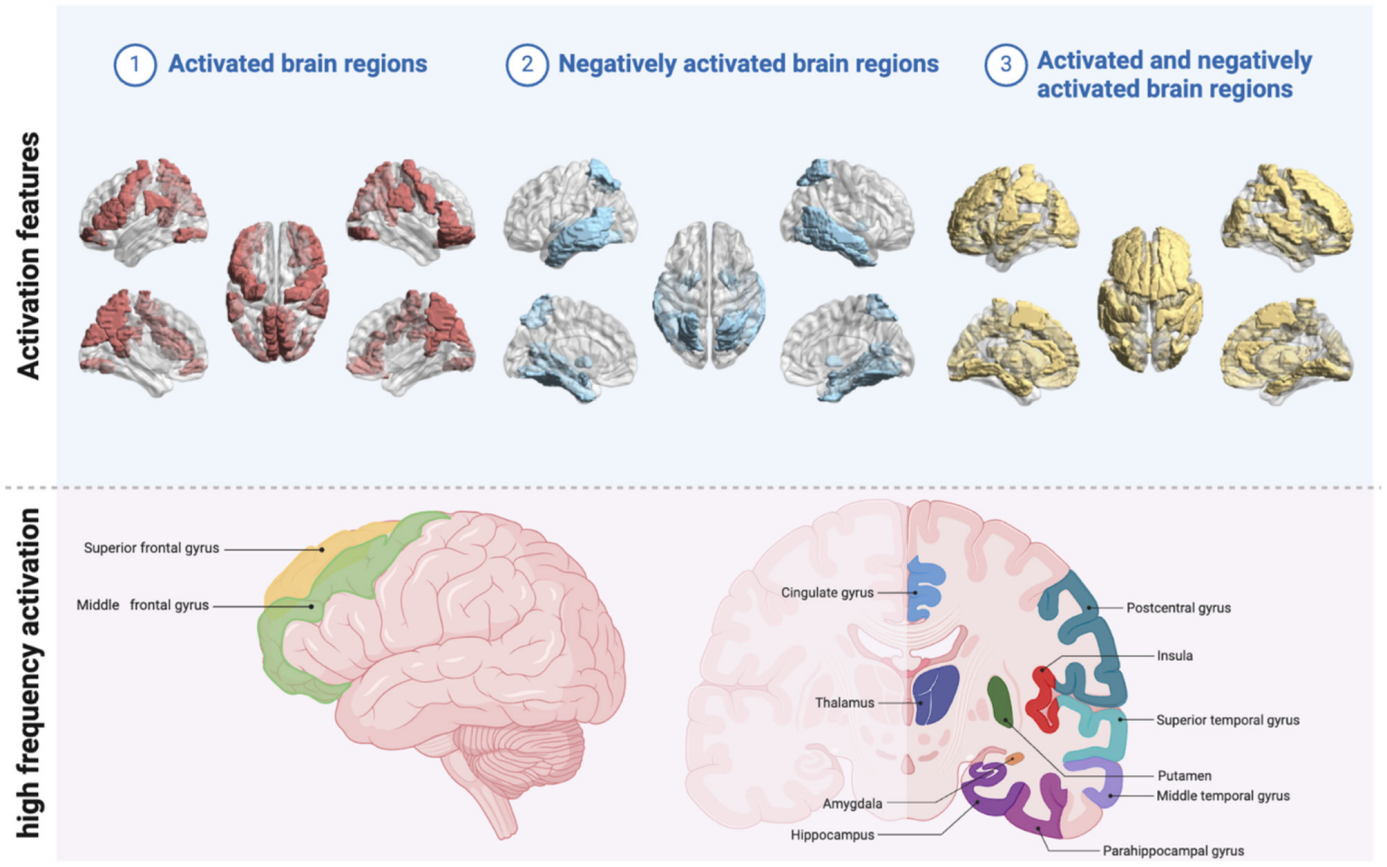
Acupuncture *deqi* activated brain regions. The light blue portion of the image above shows the overall effect of *deq*i on brain regions. Activated brain regions are shown in red. The blue parts are negatively activated brain regions. The yellow sections are brain regions with both activation and negative activation; The light pink portion of the figure below shows brain regions with high-frequency activation after *deqi* (created with BioRender.com and BrainNet).

Some studies speculate the brain central specificity of acupuncture *Deqi* by analyzing the functional relationship between the treatment of acupuncture *Deqi* and specific brain regions or brain networks. Table 2 shows that existing studies are mostly parallel controlled experiments. Compare the changes in brain activity and functional connections between brain regions activated or negatively activated by *Deqi*, such as acupuncture and sham acupuncture, sham acupuncture and no reaction, acupuncture sensation and complex sensation, different manipulation, and different acupoints. This is the main reason why brain region activation is not the same between different studies of acupuncture *Deqi*. Figure 2 shows the acupuncture *Deqi* activated, negatively activated brain regions.

**Table 2:** Study on the effect of acupuncture *Deqi* on brain center.

**Acupoints**	**Research contents**	**Instruments**	**Findings**		**References**
			**Activation**	**Negative activation**	
ST36	DM	fMRI	***Lifting-thrusting:***		Lu et al., 2017
			↑:PMv, PcG, MFG, IFG, LG, IL, Pu, CG, Cere;		
			***Twirling:***	
			↑:MOrG, MFG, STG, MOG;	↓:AWG, amygdala, ACG, ITG, MFG, AMG, PcG.	
			***Twirling plus lifting-thrusting:***	
			↑:PcG, SubG, IL, thalamus, CG, Pu;	↓:SFG, SPG, temporal gyrus, MOG, InG, LG.	
	*DQ-NR*	fMRI	S1, IL, IFG, IPL, SA, and ACG.		Jin et al., 2014
	*DQ-NR*	fMRI	LTL, IC, motor area, AMA, ACG, PCG, amygdala, hypothalamus.		Zhang, 2011
	*DQ-CS*	fMRI	SI, SII, IPL, insula, thalamus, CG, the PrCG, IFG,Pu, claustrum,STG, and the TTG and the CereVI, VIIb, VIII, CrusI, and CrusII	↓:PHG	Sun et al., 2012
	DQ-SA	TMS	↑:Motor cortex.		Sun et al., 2019
TE5	DQ-SA	fMRI	SFG, IFG, SMG, MFG, PrCG, MTG, IPL, SuTG, ITG.		Zhang et al., 2016
	DQ-SA	SPECT	↑:SMC, frontal cortex, VAC, MTG, PEC, the PA, PC, FG, IPG, PHG, LN, SN, RN;	↓:DPC, SubC.	Chen et al., 2012
	DQ-SA	PET	temporal lobe, SuTG;		Zhang et al., 2011
	DQ-SA	fMRI	PT, PO, SMG, SuTG, IC, FG, IPG, HPO, amygdala and SubN;		Lai et al., 2008
	*DQ-NR*	fMRI	↑:SuTG, MFG, ML and frontal lobe;	↓:ACL, thalamus.	Li et al., 2015
LI4	DI	fMRI	PreA, thalamus, striatum, PCG and IL.		Chen et al., 2011
	*DQ-pain sensation*	fMRI		↓:MTG, FG and LG.	Asghar et al., 2010
	AP	EEG	Alpha brain waves changed.		Yin et al., 2010
	DI	Somatosensory evoked potential		↓:N20.	Abad-Alegría and Pomarón, 2004
KI3	DQ-SA	fMRI		↓:NA, amygdala, HPO, pHPO, hypothalamus, VTA, ACG, caudate, Pu, temporal pole, and insula.	Wang et al., 2016
	DD	fMRI	***DQ combined with deep DQ:***		Bai et al., 2013
			precuneus and PCC, HPO, PCeC, and ACC;		
			***DQ combined with shallow DQ:***		
			PMv and PoC.		
	DI	fMRI	The feeling of swelling should be the best, and numbness should be avoided.		Zhu et al., 2009
BL40	PA-AA	fMRI	↑:DMN (prefrontal lobe, PCG, angular gyrus), pain matrix (SII, IL, ACG, frontal lobe, parietal lobe), CG and right Cerebellar tonsil, left hippocampal gyrus, thalamus and AMA;	↓:GP, LN, CN and left PCL.	Shi et al., 2016
	*DQ -CS*	fMRI	***Deep DQ:***		Shi et al., 2016
			↑:right PCL, left PHG, thalamus and AMA;	↓:DMN and pain matrix network.	
			***Shallow DQ:***		
			↑:right PrCG, SFG, cerebellar tonsil and bilateral thalamus;	↓:right medial prefrontal cortex.	
ST44	DQ-SA	fMRI	parietal lobe, prefrontal cortex and PIA;		Usichenko et al., 2015
LR3	*DQ -pain sensation*	fMRI	somatosensory and TIC.		Fang et al., 2012
TE5	DQ-SA	fMRI	↑:SI, SMC, frontal cortex, DPC, Frontopolar area, Orbitofrontal area, IC, ITG, MTG, FG, Angular gyrus, SMG, Subcentral area and IPG, the head of PHG, thalamus and RN;	↓:SI, PMC, SAC, SMC, DPC, Frontopolar area, VAC,VACC,DPCC,SMG and DPC.	Huang et al., 2013
SP6	*DQ-NR*	Viking Quest	The relative latency of P60-N75 in the DQ group was significantly shorter than that in NR group.		Lin et al., 2015
TE5,ST36	PA-AA	fMRI	↑:IPL, SWM, SuTG, GFM, prefrontal lobe, cuneate lobe, PcG, VDC, mesencephalon, precuneus, COG, FLFG, SMG;	↓:PCG, Pu, IPL, culmen cerebelli, intercerebral fissure, clivas, thalamus, CG, occipital lobe, SuTG, GFM, dentate body of Cere, corpus callosum, midtemporal gyrus, IL, mesencephalon, subthalamic nucleus.	Tian et al., 2014
ST36, ST39	*DQ-NR*	fMRI	CG, IL, superior wall of lateral sulcus and PcG.		Gong et al., 2003
ST36,LI4, LR3	DI	fMRI	↑:Sensorimotor, parietal lobe;	↓:MPL, MPaL and MTL.	Hui et al., 2009
TE5,LR3	DQ-SA	fMRI	↑:SI, SMC, frontal cortex, Frontopolar area, IC, ITG, MTG, SuTG, Ventral ACC, FG, Angular gyrus, SMG, IPG, PCL, semilunar lobule, sub-lobar, CN, limbic lobe, HPO, PHG, thalamus, midbrain RN, brain stem, cerebellar tonsil;	↓:SI, PMC, SAC, SMC, SAC, DPC, Frontopolar area, VAC, SMG, ACL, culmen.	Lin et al., 2015
TE5, PC6	DQ-SA	fMRI	DMN.		Zhang et al., 2016
CV12,LR3	DI	fMRI	↑:left MPS, left TPS and right SCS;	↓:ACG and MTC, left SFG, left straight gyrus, right orbital gyrus and sulcus, right SuTG and sulcus, right temporal pole and right anterior segment of cricoid sulcus.	Wang et al., 2013
HT5, GB39	Acupoint and non-acupoints	fMRI	SI and language-related cortex.		Xiao et al., 2016
CV4, CV12	DA	fMRI	MFG, ACG, ICG, HPO.		Fang et al., 2011

DM, Different manipulation; DQ, Deqi; NR, no reaction; CS, complex sensation; SA, Sham Acupuncture; DI, Different intensity; AP, Acupuncture process; DD, Different depth; PA, Before Acupuncture; AA, after Acupuncture; DA, Different acupoints; PMv, premotor cortex; PcG, postcentral gyrus; MFG, middle frontal gyrus; IFG, inferior frontal gyrus; LG, lingual gyrus; IL, insular lobe; Pu, putamen; CG, cingulate gyrus; Cere, cerebellum; HPO, hippocampus; CauG, caudate gyrus; MOrG, middle orbital gyrus; STG, subfrontal triangular gyrus; MOG, middle occipital gyrus; AWG, anterior wedge gyrus; ACG, anterior cingulate gyrus; ITG, inferior temporal gyrus; AMG, auxiliary motor gyrus; SubG, suboccipital gyrus; SFG, superior frontal gyrus; SPG, superior parietal gyrus; InG, insular gyrus; IPL, inferior parietal lobule; SA, shielding area; LTL, Left temporal lobe; IC, insular cortex; AMA, auxiliary motor area; PCG, posterior central gyrus; SI, somatosensory cortex; SII, secondary somatosensory cortex; PrCG, precentral gyrus; SuTG, superior temporal gyrus; TTG, transverse temporal gyrus; PHG, parahippocampal gyrus; SMG, supramarginal gyrus; MTG, middle temporal gyrus; SMC, Supplementary Motor Cortex; VAC, Visual Association Cortex; PEC, Posterior Entorhinal Cortex; PA, pregenual area; PC, Perirhinal Cortex; FG, Fusiform gyrus; IPG, Inferior prefrontal gyrus; LN, lenticular nucleus; SN, screen nucleus; RN, red nucleus; DPC, Dorsolateral prefrontal cortex; SubC, Subgenual cortex; PT, pars triangularis; PO, pars opercularis; SubN, substantia nigra; MFG, medial frontal gyrus; ML, marginal lobe; ACL, anterior cerebellar lobe; PreA, Prefrontal area; PCC, posterior cingulate cortex; PCeC, posterior central cortex; ACC, anterior cingulate cortex; PoC, postcentral cortex; NA, Nucleus accumbens; pHPO, parahippocampus; VTA, ventral tegmental area; GP, Globus pallidus; LN, lentiform nucleus; CN, caudate nucleus; PCL, posterior cerebellar lobe; DMN, default mode network; PIA, posterior insular area; TIC, thalamic insular cortex; SWM, subcortical white matter; GFM, gyrus frontalis medius; VDC, ventriculus dexter cerebri; COG, central occipital gyrus; FLFG, frontal lobe frame gyrus; PMC, Primary Motor Cortex; SAC, Somatosensory Association Cortex; DPCC, Dorsal Posterior cingular cortex; VACC, Ventral anterior cingulate cortex; MTC, medial temporal cortex; ICG, inferior cingulate gyrus; MPL, Medial prefrontal lobe; MPaL, medial parietal lobe; MTL, medial temporal lobe; MPS, medial parietal sulcus; TPS, transverse parietal sulcus; SCS, superior cricoid sulcus;↑: Activation;↓: Negative activation.

**Figure 3:**
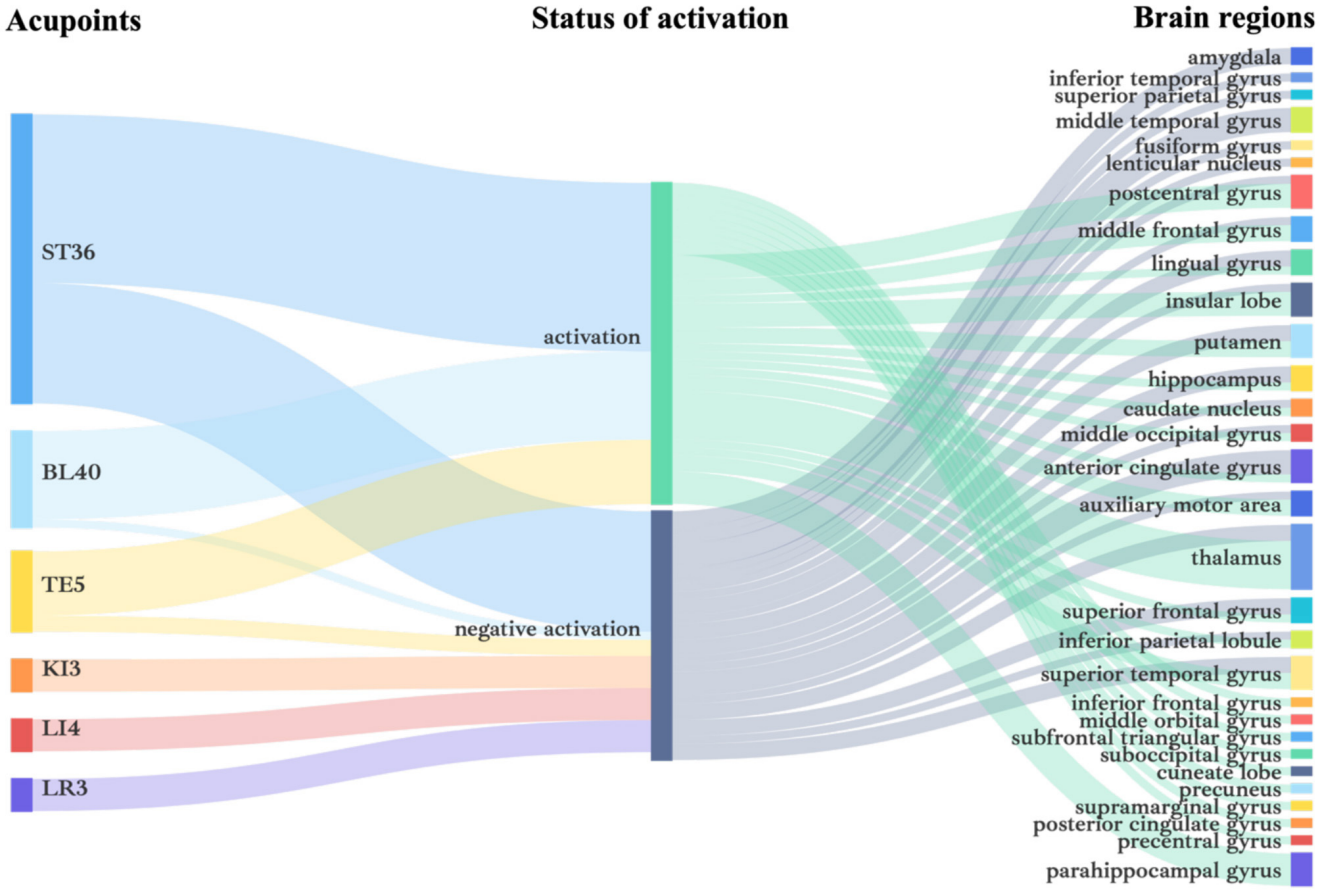
Acupoints and brain region activation characteristics in studies of acupuncture *Deqi*. The analytical form of Sankey diagrams is used to show the interactions and composition of acupuncture *deqi* between acupoints, brain regions, and activation situations.

Among them, the middle temporal gyrus, cingulate gyrus, amygdala, hippocampus, superior frontal gyrus, and superior temporal gyrus were the most mentioned negatively activated brain regions. The more recognized activated brain regions were the parahippocampal gyrus, postcentral gyrus, insular, middle frontal gyrus, and superior temporal gyrus. It is noteworthy that brain regions such as putamen, thalamus, etc. showed different activation states in different studies (Figure 3). It is evident that the central brain mechanism of *Deqi* is still controversial and needs to be further explored through rigorous experimental design.

Several results have shown that compared with sham acupuncture, non-acupoints acupuncture or no reaction acupuncture, acupuncture *Deqi* can cause more stable and extensive activation of the brain area, and the degree of *Deqi* sensation is in direct proportion to the degree of brain activation (Lai et al., 2008; Hui et al., 2009; Li et al., 2015; Usichenko et al., 2015; Xiao et al., 2016; Sun et al., 2019). Most of the selected acupoints are commonly used in the limbs, and the frequency from high to low is ST36, BL40, TE5, KI3, LI4, LR3 (Figure 3). Effects of Acupuncture may be achieved through the meridian network-cerebral cortex-organs, but the central brain afferent pathways are different for different acupoints, which is related to the fact that acupuncture different acupoints produces relatively specific brain activities (Fang et al., 2011).

However, Gong et al. found that there was no significant difference in the localization of brain functional activity areas after acupuncture ST36 and ST39 respectively (Gong et al., 2003). This may be because the therapeutic effects of acupoints are similar, indicating that the efficacy of acupoints is closely related to the regulatory function of activated brain regions. It has been found that *Deqi* sensation and pain sensation cause different brain area changes. Fang et al. showed that acupuncture generates strong and extensive negative activation regions in the limbic-paralimbic-neocortical-network (LPNN) during the process of *Deqi*, and enhances the brain functional network connections in the negatively activated brain regions. When acupuncture causes pain, the degree of negative activation in the brain region decreases and the area shrinks, and most of the fMRI signals in the brain area are reversed into activation signals. The functional network between brain regions activated by pain is significantly enhanced, resulting in the functional network effect antagonistic to *Deqi*, which may be related to the central mechanism of acupuncture analgesia (Zhu et al., 2009; Fang et al., 2012; Sun et al., 2012; Wang et al., 2013). Therefore, in the process of acupuncture, we should try to avoid the generation of bad stimulation such as tingling.

In addition, different acupuncture manipulations can produce different changes in brain regions. Studies have shown that deep acupuncture stimulates more network connections between brain regions (Shi et al., 2016). Lifting-thrusting, twirling, and twirling plus lifting-thrusting activated areas related to the somatosensory system, vision, cognition, and emotion regulation. The strongest signals were activated by lifting-thrusting, especially in the LPNN region, followed by twirling plus lifting-thrusting, while twirling was the weakest (Lu et al., 2017).

Existing studies have pointed out that LPNN plays an important role in the regulation of acupuncture *Deqi*. This network plays a central regulatory role in the process of human cognition, emotion, memory regulation, and internal environment stability (Fang et al., 2009). In 2000, Hui Kathleen KS applied fMRI technology to find that acupuncture *Deqi* can negatively activate the limbic lobe and subcortical gray matter structure (Hui et al., 2000). With the development of neuroscience, this theory has been confirmed in many subsequent studies, and the hypothesis of “acupuncture modulation LPNN” has been developed (Fang et al., 2009). This hypothesis provides a strong scientific basis for acupuncture therapeutic brain mechanisms.

Other scholars put forward the hypothesis of “meridian-brain correlation” and redefined the concept of *Deqi* as “the reaction in brain region after acupuncture intervention through brain integration”, which is different from other physiological and pathological reactions in the brain and is the most important sign that acupuncture has “therapeutic effect” on the level of central adjustment (Lai and Huang, 2007). Then fMRI was used to observe the difference between *Deqi* and no action by acupuncture at TE5. It was found that there is a specific direction in the brain region during *Deqi*, which confirms its hypothesis to a certain extent and defines the concept of *Deqi* (Lai et al., 2008). This provides an objective basis at the central level for confirming the acupuncture effect promoted by acupuncture *Deqi* and the specificity of the acupoint effect, which is helpful to summarize the rules of acupoint specificity in central effect and make the clinical selection of acupoints more accurate, to improve the clinical efficacy of acupuncture. Some progress has been made in the application of brain function detection techniques such as fMRI in the research of acupoint specificity and acupuncture mechanism, and it has become an important diagnostic method to unveil the mystery of acupuncture.

#### 2.1.3 Target organ mechanisms of acupuncture Deqi

The final link of acupuncture is to act on the effect target organ, correct the abnormal state of the target organ, and achieve the effect of treating the disease. Table 3 lists in detail the relevant studies on the effect of acupuncture *Deqi* on target organs. These studies focused on normal individuals and patients with coronary heart disease, observing changes in indicators such as heart rate, blood pressure, and gastric electricity after acupuncture *Deqi*, involving acupoints including ST36, PC6, LI4, and LI11.

**Table 3:** Study on the effect of acupuncture *Deqi* on target organ.

**Acupoints**	**Research content**	**Instruments**	**Results**	**References**
ST36	DM	Electrocardiograph	***Lifting-thrusting and twirling:*** blood pressure and heart rate↓.	Huang et al., 2012c
ST36, PC6	*DQ-NR*	Impedance blood-diagram-scope, Gastrointestinal electrograph	Change gastroelectric wave parameters.	Huang, 1999
PC6	*DQ-CS*	Electrophysiolograph	Change cardiac output SV, CO, ST segment, and electrocardiogram T-wave.	You et al., 1992
LI4, LI11	DQ-SA	Blood pressure monitor, Electrocardiogram	Heart rate, mean arterial pressure and LF/HF values of heart rate variability↑.	Yu and Jones, 2013

DM, Different manipulation; DQ, Deqi; NR, no reaction; CS, complex sensation; SA, Sham Acupuncture.

In research conducted by Huang et al. found that both the lifting-thrusting and twirling of acupuncture with ST36 can effectively reduce blood pressure and heart rate in normal individuals, and improve heart rate variability (Huang et al., 2012c). Yu et al.'s study also found similar results. Compared with sham acupuncture, acupuncture with LI4 and LI11 can significantly regulate heart rate variability, mean arterial blood pressure, and heart rate in normal individuals, and the intensity of *Deqi* acquisition increases with the increase of sympathetic discharge in the autonomic nervous system (ANS) (Yu and Jones, 2013). Some authors believed that *yang* is the sympathetic division of the ANS (Tjen-A-Looi et al., 2004). Acupuncture stimulation can regulate the cerebellar limbic system to cause changes in the autonomic nervous system (Ueyama, 2012). Since ST36, LI4, and LI11 are all located on the *yang* meridian, stimulating these acupoints may induce changes in the ANS by regulating brain activity, thereby affecting the physiological function of target organs in the body.

The two studies included in this review demonstrate the acupoint therapeutic functions of ST36 and PC6, such as “ST36 for treating gastrointestinal diseases” and “PC6 for treating heart disease, with the effect of calming the heart and mind”. Huang's research on normal individuals found that acupuncture with ST36 can alter gastric electrical wave parameters (You et al., 1992). Recent studies have also shown that compared to sham acupuncture, acupuncture ST36 can improve gastrointestinal peristalsis, which is an alternative treatment for gastrointestinal spasms (Vieira et al., 2017). Another clinical study found that percutaneous acupoint electrical stimulation of ST36 can improve chronic constipation, which may be mediated by enhancing parasympathetic nerve activity (Xiao et al., 2022). Huang and You's studies both focused on patients with coronary heart disease and observed that acupuncture with PC6 can improve multiple cardiac function indicators (You et al., 1992). Recent studies have confirmed that PC6 plays a crucial role in cardiovascular disease, which may be achieved by regulating various signals of mitochondrial energy metabolism (Chen M. et al., 2019).

### 2.2 Animal experimental research

To further delve into the mechanisms of acupuncture *Deqi*, more and more studies are focusing on animal experiments. For ethical and technical reasons, it is not possible to observe the physiological mechanisms of acupuncture *Deqi* in humans. Animal experiments, on the other hand, provide a good platform environment for studying acupuncture *Deqi*.

The effect of acupuncture *Deqi* was similarly verified in animal experiments (Table 4). Acupuncture *Deqi* was able to reduce the release of local inflammatory mediators and decrease the pain-allergic reaction caused by sympathetic efferents (Li and Shi, 2001). In a rat model of dysmenorrhea, acupuncture *Deqi* was able to enhance the expression of opioid receptors in the midbrain and spinal cord to provide analgesia (Qi et al., 2015b). Several studies have demonstrated that acupuncture *Deqi* can improve uterine microcirculatory disorders and alleviate uterine contractions, with significant efficacy compared to the no-*Deqi* group (Shen et al., 2014; Qi et al., 2015a).

**Table 4:** Study on the effect of acupuncture *Deqi* on animal experiments.

**Acupoints**	**Animals**	**Instruments**	**Findings**	**References**
ST36	hamsters	light microscope electron microscope	The biological basis of *Deqi* may be mediated by connective tissue.	Shi and Zhang, 1996
SP6	SD rats	RT-PCR	*Deqi* exerts analgesic effects by enhancing opioid receptor expression in the midbrain and spinal cord.	Qi et al., 2015d
SP6	SD rats	Cold light source microcirculation microscope	Deqi significantly improves uterine microcirculation.	Shen et al., 2014
SP6	SD rats	Cold light source microcirculation microscope	*Deqi* relieves uterine contractions and has some follow-through effects.	Qi et al., 2015c
DU14	SD rats	Electronic digital thermometer	One of the objective features of *Deqi* is an elevated temperature in the tail of the rats.	Lv et al., 2013
GV14	SD rats	Morris Water Maze HE staining Western blot	*Deqi* can better promote Aβ transporter and degradation, thus reducing brain Aβ level and improving cognitive function.	Lv et al., 2022
ST36,ST39	rabbit	myoelectricity	The sensation of sinking tightness under the acupuncturist's hands was accompanied by EMG issuance at the acupoints.	Zeng et al., 1982
GB34,GB36	Wistar rats	Spontaneous Pain Behavioral Response Skin flap immersion	*Deqi* is able to reduce the release of local validating mediators and sympathetic efferents resulting in pain hypersensitivity.	Li et al., 2001
ST36,CV12	SD rats	pyloric intramural balloon	*Deqi* may exert a significant modulatory effect on gastric motility by activating afferent fibers.	Su et al., 2014

## 3 Limitations and perspectives relating *Deqi* studies

In conclusion, acupuncture *Deqi* has always been one of the focuses of research in traditional medicine. The strength of the “*Deqi*” feeling should be based on the comfort of the patient (Tian et al., 2021). Excessive or over-stimulation can destroy the therapeutic effects of acupuncture. If *Deqi* feeling is not enough, it will not be able to achieve the effect of boosting and regulating qi. It can be seen that *Deqi* is a key factor in the effectiveness of acupuncture. How to accurately select acupoints, choose acupuncture techniques, and grasp the strength of *Deqi* in clinical application is the key and difficult point of acupuncture treatment. *Deqi by* acupuncture stimulation is the comprehensive manifestation of “acupuncture”, “acupoint”, “sensation”, “acupoint effect”, etc. The core problem of acupuncture *Deqi* is how to apply modern scientific methods to confirm its theory and reveal its physiological mechanisms. Therefore acupuncture *Deqi* urgently needs to carry out objective quantitative experimental research. However, many factors such as the status of the research object, the choice of the *Deqi* scale, the design of the experimental scheme, the manipulation of the acupuncturist, and the stimulation parameters will affect the accuracy of the research results. Quantitative studies of acupuncture *Deqi* need to be further optimized in terms of methodology and clinical validation.

### 3.1 The mechanism of *Deqi* is closely related to the brain, but remains controversial

The transmission of information in acupuncture *Deqi* is closely related to brain centers. With the development of science and technology, researchers can study the structural and functional changes in the brain after acupuncture *Deqi* under noninvasive conditions. Various studies have explored the central brain mechanisms of acupuncture *Deqi* through different testing techniques and have achieved some results. Among them, fMRI was the most used testing tool due to its high spatial resolution, non-invasiveness and flexibility (Fukuda et al., 2021). Also using fMRI as a testing modality, acupuncture manipulation of a single acupoint revealed that *Deqi* was able to activate specific brain regions. For example, needling ST36 to produce the sensation of *Deqi* can activate brain regions such as the inferior frontal gyrus, insular lobe and cingulate gyrus. This was consistent with the results of existing studies on the mechanism of acupuncture (Huang et al., 2022).

However, some controversial results have been found in the acupuncture *Deqi* literatures. For example, needling ST36 produces *Deqi* sensations when putamen is activated. In contrast, putamen is negatively activated when other points are used. Superior temporal gyrus was activated in studies applying TE5, but had a different activation state in studies applying other acupoints. Similarly, thalamus was activated in the study after needling BL40, but not otherwise. It is hypothesized that the activation of brain regions in acupuncture *Deqi* is related to the choice of acupoints. According to Traditional Chinese Medicine (TCM) theory, the therapeutic effect of an acupoint is specific. This is related to the function of the viscera and organs responsible for the meridian to which it belongs (Rong et al., 2011). It has been shown that acupoints where acupuncture improves visual and auditory functions can correspond to the activation of the visual and auditory cortex (Li et al., 2003; Parrish et al., 2005). However, whether this is a general property of acupuncture *Deqi* needs further confirmation.

In addition, acupuncture *Deqi* studies have identified a large number of negatively activated brain regions such as the middle temporal gyrus, cingulate gyrus, amygdala, lingual gyrus, hippocampus, thalamus, superior frontal gyrus, the superior parietal gyrus and fusiform gyrus. The above brain regions belong to the LPNN and limbic system. This is similar to the results of previous studies on the effects of acupuncture on brain centers (Wu et al., 1999; Bai et al., 2009; Asghar et al., 2010; Liu et al., 2011). The LPNN is involved in processing such as analgesia, anxiety relief, and improvement of cognitive function (Fang et al., 2009). The functions of the limbic system involve improving pain, regulating mood, controlling sleep activity, and participating in memory activity (Murray et al., 2021; Alvarez-Salas et al., 2022; Kamali et al., 2023). And how acupuncture *Deqi* works by negatively activating the LPNN and limbic system to produce clinical efficacy needs further study.

In addition to the choice of acupuncture points, different acupuncture manipulations can also have an effect on deqi. During acupuncture, it is often necessary to produce deqi sensations by lifting-thrusting or twisting the needle up and down (Zhao, 2008). Specifically, they include twirling and lifting-thrusting. Lu et al. stimulated at ST36 using 3 types of acupuncture manipulations: twisting, lifting-thrusting, and twisting plus lifting-thrusting. The study found that although the activated brain regions did not completely overlap, they equally activated regions associated with the somatosensory system, vision, cognition, and emotion regulation. In terms of the degree of deqi, the MASS index showed that lifting-thrusting > twirling plus lifting-thrusting > twirling (Lu et al., 2017). The results of another study also confirmed that the deqi sensation produced by lifting-thrusting was more intense than that produced by twisting (Huang et al., 2012c). Huang et al. also corroborated the difference in the degree of deqi produced by different acupuncture manipulations from the perspective of body temperature. Compared with twisting, lifting-thrusting can cause a more pronounced increase in body temperature (Huang et al., 2013).

However, most of the existing studies were conducted on healthy volunteers, and the results were less reproducible and consistent. On the one hand, existing studies have different experimental designs, subjects, stimulation methods, and *Deqi* evaluation methods, which make the results of different studies different and are not conducive to combinatorial analyses. On the other hand, experimental image processing techniques, such as processing accuracy and brain segmentation methods, vary in functional magnetic resonance imaging studies. The above reasons pose certain problems in revealing the biological mechanisms behind the *Deqi* phenomenon.

### 3.2 Acupuncture *Deqi* can work by affecting “local acupoints–brain–target organs”

Most of the studies on local and nearby acupoints focused on the local material structure, temperature, luminescence, and heat generation. The local tissue distribution of *Deqi*'s acupoints is dominated by muscle fibers and nerve endings, with higher densities of Ca^2+^ and mast cells also present. The basis of *Deqi* lies in the contraction of muscle fibers triggered by needling, the electrical conduction of nerves, the release of locally active substances, and the transmission of information between cells. To a certain extent, the results of these studies responded to the phenomenon of *Deqi* with changes in material and energy metabolism, but potential mechanisms relating to *Deqi* by acupuncture are still challenging.

Target organ testing refers to the observation of *Deqi* mechanisms by detecting functional changes in acupoint-related or disease-related target organs before and after acupuncture. The number of studies that have been conducted using target organs as effect entry points is small and includes a limited variety of target organs. Multiple indicators should be fully utilized in conjunction with the characteristics of the disease, acupoints, and techniques to expand the types of target organs studied. *Deqi* is a key factor affecting the therapeutic effect of acupuncture, and the *Deqi* effect is positively correlated with the therapeutic effect (Takahashi, 2011). However, there are relatively few studies on acupuncture *Deqi* in disease states This requires both normal individuals and patients as research subjects to compare the differences in changes in target organs after acupuncture at specific acupoints for *Deqi*. In addition, it is recommended to cross-fertilize the techniques of acupoint localization, brain center, and target organ to carry out the real-time dynamic study of acupuncture *Deqi*.

**Figure 4:**
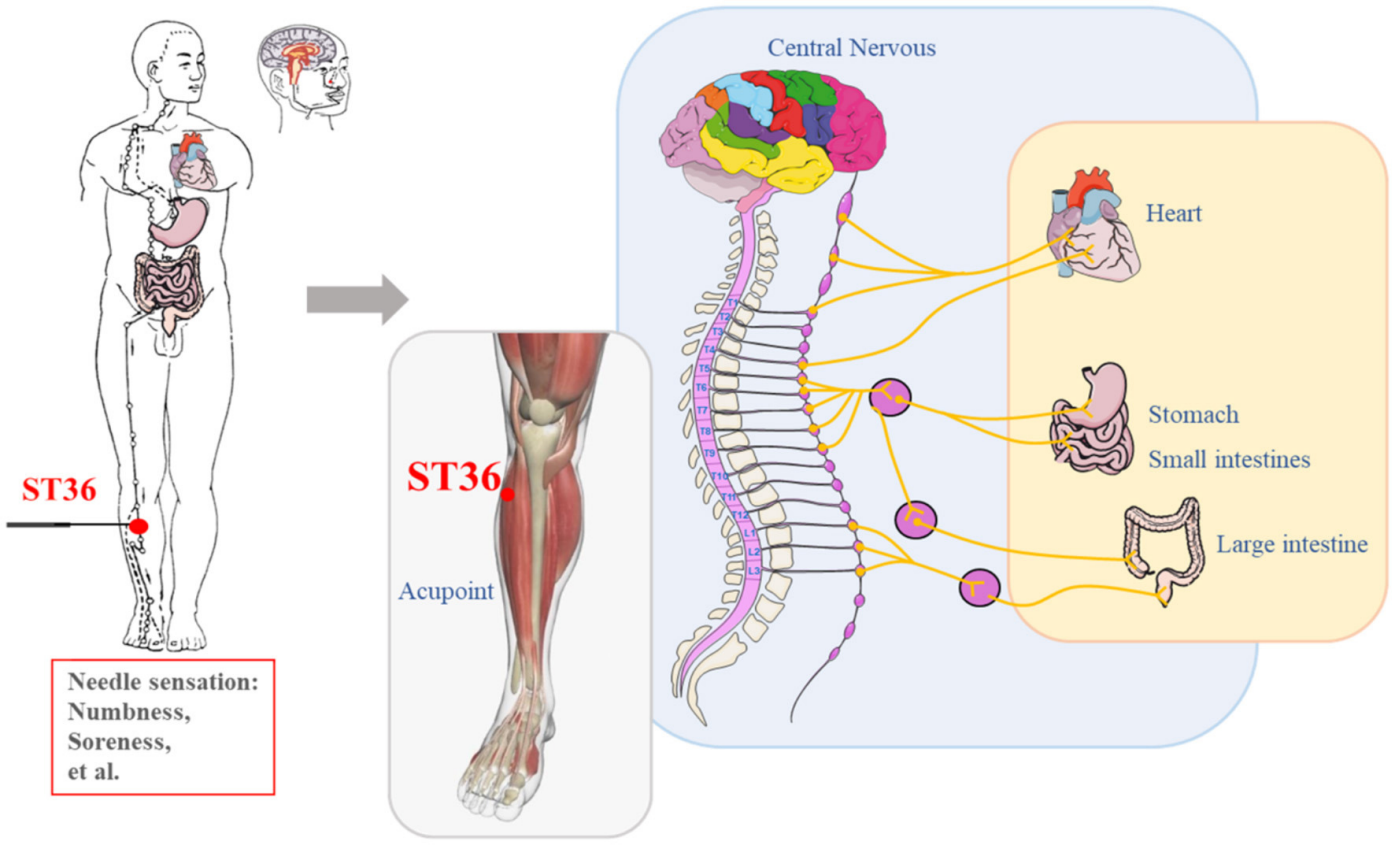
An example of applying acupuncture at ST36 to produce *Deqi* affects multiple systems of the body.

In summary, acupuncture *Deqi* is achieved by stimulating local acupoints, affecting biological signals such as skin temperature, blood flow velocity, and muscle contraction. These signals are then uploaded from the periphery to the central nervous system, activating or inhibiting the functional activities of the corresponding cerebral cortex such as LPNN, generating *Deqi* sensation, and finally acting on the target organ to achieve therapeutic effects (Figure 4). In the process of acupuncture Deqi, the local acupoint stimulation end, the brain is the integration end, and the target organ is the effect end. By needling localized acupoints *Deqi* sensations are induced and these *Deqi* sensations are transmitted to the brain to be further processed. Finally the abnormal state of the target organ is corrected.

Questions about the physiological mechanisms of acupuncture *Deqi* still deserve further exploration. Acupuncture can cause an increase in the expression of calcitonin gene related peptides and substance P in the skin, and the degranulation of mast cells in the cortex of the acupoint area releases a large amount of histamine and 5-hydroxytryptamine. It is speculated that these active substances may activate various afferent nerve endings in the cortex, causing changes in skin temperature, resulting in needle sensation and transmission of needle effect (Wu et al., 2015). The *Deqi* sensation produced by acupuncture often leads to muscle contraction in the rich areas, which may be mediated by ASIC3 and TRPV1 channels in the muscles (Ugawa et al., 2002; Deval et al., 2010). The local biological and chemical signals of acupoints may be transmitted to the central nervous system through the mediation of the sympathetic nervous system. Acupuncture can also activate various groups of nerve fibers in the acupoint area to transmit electrical signals to the central nervous system, and different types of sensory signals are transmitted from different types of fibers (Wang et al., 1985; Sato et al., 1992, 1993). The signals generated by acupuncture *Deqi* are integrated through the central nervous system to regulate different brain functional activities, depending on the different therapeutic characteristics of acupoints, and the changes in these brain regions are different from sensations such as pain and touch. The functional changes in the brain may regulate the physiological functions of acupoint-related or disease-related target organs by inducing the autonomic nervous system.

Although mechanistic studies of acupuncture *Deqi* involve the whole process of Deqi sensation, these studies still have some limitations. The local acupoint is the initial site for triggering the sensation of *Deqi*, the brain is the advanced center for producing the *Deqi* sensation and the target organ is a manifestation of therapeutic changes. All three are closely related to the generation of the *Deqi* sensation. However, current research mainly focuses on exploring the changes in the local acupoint, brain function, and target organ, without simultaneously observing the changes in all three. This may be due to the limitations of the state of the art to conduct a multidisciplinary, multi-targeted, dynamic, whole-process study of acupuncture *Deqi*. In the future, more clinical and basic studies based on physiological and disease states from multiple perspectives are needed to reveal in depth how the material and energy basis of *Deqi* by acupuncture is transmitted to the central nervous system, integrates brain functions, and ultimately acts on the target organs. Clarifying the relationship between *Deqi* and curative effect is of great significance to the in-depth exploration of acupuncture theory and practice, and to promote the international development of acupuncture.

## Author contributions

ZZ: Conceptualization, Visualization, Writing – original draft. LY: Conceptualization, Visualization, Writing – original draft. Y-ZL: Data curation, Investigation, Writing – original draft. YW: Data curation, Investigation, Writing – original draft. MH: Project administration, Writing – review & editing. M-MS: Project administration, Writing – review & editing. H-PH: Supervision, Writing – review & editing. S-QM: Formal analysis, Writing – original draft. H-ZZ: Formal analysis, Writing – original draft. M-YL: Methodology, Writing – original draft. X-YZ: Methodology, Writing – original draft. D-YC: Funding acquisition, Writing – review & editing. H-FW: Funding acquisition, Writing – review & editing.
